# Exploring the Perspectives of Unhoused Adults and Providers Across the HCV Care Continuum

**DOI:** 10.1177/10547738241273104

**Published:** 2024-09-09

**Authors:** Benissa E. Salem, Helena Almeida, Sarah Akure Wall, Kartik Yadav, Alicia H. Chang, Lillian Gelberg, Adeline Nyamathi

**Affiliations:** 1 University of California, Los Angeles, USA; 2 University of California, Irvine, USA; 3 Los Angeles County Department of Public Health, CA, USA

**Keywords:** people experiencing homelessness, health and social service providers, HCV, HCV care continuum

## Abstract

Hepatitis C virus (HCV), the most common blood-borne infection, disproportionately affects people experiencing homelessness (PEH); however, HCV interventions tailored for PEH are scarce. This study utilized a community-based participatory approach to assess perceptions of HCV treatment experiences among HCV-positive PEH, and homeless service providers (HSP) to develop and tailor the “I am HCV Free” intervention which integrates primary, secondary, and tertiary care to attain and maintain HCV cure. Four focus groups were conducted with PEH (*N* = 30, *M*_age_ = 51.76, standard deviation 11.49, range 22–69) and HSPs (*n* = 10) in Central City East (Skid Row) in Los Angeles, California. An iterative, thematic approach was used to ensure the trustworthiness of the data. Barriers and facilitators emerged from the data which have the potential to impact initiating HCV treatment and completion across the HCV care continuum. Understanding and addressing barriers and strengthening facilitators to HCV treatment will aid in HCV treatment completion and cure for PEH.

## Introduction

Hepatitis C virus (HCV), a common blood-borne infection, disproportionately impacts people experiencing homelessness (PEH) (Williams & Bryant, 2018). One of the major HCV risk factors among PEH includes injection drug use (Fokuo et al., 2020), needle sharing, and acquiring tattoos from unregulated settings (Schillie et al., 2020). By 2030, the World Health Organization (WHO) has aimed to eliminate HCV infection (WHO, 2022; Wuerth et al., 2020). While early detection and treatment are keys to preventing the spread of HCV, half of HCV-positive PEH are unaware of their infection (Gelberg et al., 2012; Strehlow et al., 2012) which impacts the HCV care continuum with respect to screening, diagnosis, linkage to care, treatment, and cure (Meyer et al., 2015).

### HCV Care Continuum Barriers Among PEH

Along the care continuum, HCV-positive PEH may experience a lack of HCV knowledge regarding screening, transmission, diagnosis, and treatment availability (Masson et al., 2020). HCV screening is impacted by HCV stigma (Marinho & Barreira, 2013). Lack of chronic disease self-care management for mental health/psychiatric conditions likewise inhibits HCV treatment (Masson et al., 2020). HCV-positive PEH with drug dependence may be unable to manage withdrawal symptoms, control drug use (Masson et al., 2020), and HCV medication adherence concurrently. Devoid of proper linkage to HCV care, it is difficult for PEH to adhere to HCV treatment (Benitez et al., 2020). In fact, lack of insurance access and coverage (Fokuo et al., 2020) impacts HCV linkage to care. Without access to HCV diagnosis and treatment, liver fibrosis may result which can lead to further complications (i.e., ascites, hepatic encephalopathy, etc.) (Huizen, 2019; Nall, 2018).

### HCV Care Continuum Facilitators Among PEH

Despite HCV treatment barriers, HCV medication treatment facilitators among PEH include individual-level and system-level factors. Individual-level facilitators include internal motivation (Beiser et al., 2017; Masson et al., 2020) and a desire to maintain health. System-level facilitators include shelter partnerships (Fokuo et al., 2020) and access to case management (Beiser et al., 2017). In addition, needle exchange programs are highly effective in reducing HCV infection (Abdul-Quader et al., 2013). There has been a high success in HCV treatment completion with the use of coordinated programs that support screening, and linkage to care using a trained HCV care coordinator who manages treatment paperwork, treatment procurement, and provides HCV treatment support (Benitez et al., 2020).

### Evolution of HCV Treatment, Advent of DAAs, and Cure

Over the past three decades, there has been rapid development and evolution of HCV treatment from recombinant interferon-alfa (Caroll, 2019) to oral direct-acting antivirals (DAAs). Compared to past HCV medication regimens, DAAs are used to treat HCV infections with shorter treatment duration, fewer side effects, and higher sustained virologic response (SVR) (Caroll, 2019). However, DAAs may be limited in many areas. Providing low threshold access to treatment and adherence support may be two approaches to attaining HCV cure (Harris & Rhodes, 2013).

### Application of CHW-Delivered, Nurse-Led Interventions to the “I am HCV Free” Intervention

Several studies conducted for nearly the past 10 years have utilized the registered nurse (RN)/community health worker (CHW) model to provide preventive treatments combined with education such as hepatitis A virus/hepatitis B virus vaccination, latent tuberculosis medication adherence (Nyamathi et al., 2015, 2022; Nyamathi, Wall et al., 2021; Nyamathi, Salem et al., 2021; Salem et al., 2020) and decrease frailty and drug and alcohol use (Salem et al., 2017) for PEH and those who have histories of incarceration. However, few have been designed to test the RN/CHW intervention on HCV medication delivery among PEH.

The “*I am HCV Free*” intervention was designed to be delivered by an RN/CHW team (Nyamathi et al., 2021). Once participants are enrolled in the intervention, they will complete once weekly eight individual education sessions focused on personal goals and values, the impact of drug and alcohol use, basic problem-solving, and reducing HCV/human immunodeficiency virus (HIV) risk (Nyamathi et al., 2021). Once a week, the RN/CHW team would deliver daily directly observed HCV therapy to participants and provide them with health and social service referrals (Nyamathi et al., 2021). By contrast, participants enrolled in the 8-week, clinic-based, standard of care receive their DAA medication in a clinic, however, do not receive HCV education sessions delivered by an RN/CHW team or accompaniment to needed services (Nyamathi et al., 2021).

### Comprehensive Health Seeking and Coping Paradigm: A Guiding Theoretical Framework

This study was informed by the Comprehensive Health Seeking and Coping Paradigm (CHSCP) (Nyamathi et al., 1989). The CHSCP helped to shape the semi-structured interview guide (SSIG) and considered the situational and personal factors, resources, health-seeking and coping behaviors of PEH, as well as their perceptions related to their compliance with HCV medication, along with their immediate and long-term health goals (Nyamathi et al., 1989).

The CHSCP has guided numerous studies (Nyamathi et al., 2015, 2018, 2021, 2022) which have developed and implemented interventions for PEH. In this study, the SSIG prompted questions such as (a) “What health services you have sought in the last few years?” (b) “If you ever had a time when you had a health problem but did not seek care for it, what got in the way?” (c) “What would you say are the biggest barriers to completing the therapy (HCV)?” and (d) “What would you recommend to successfully engage PEH to complete the testing process?”

### Purpose

This paper is part of a two-part qualitative series (Nyamathi et al., 2021) which had a common purpose; however, each paper uniquely contributes to the understanding of HCV treatment experiences among HCV-positive PEH and homeless service providers (HSP, e.g., CHWs, RNs, Chaplains) to tailor the “I am HCV Free” intervention for PEH.

## Methods

**Figure 1.**
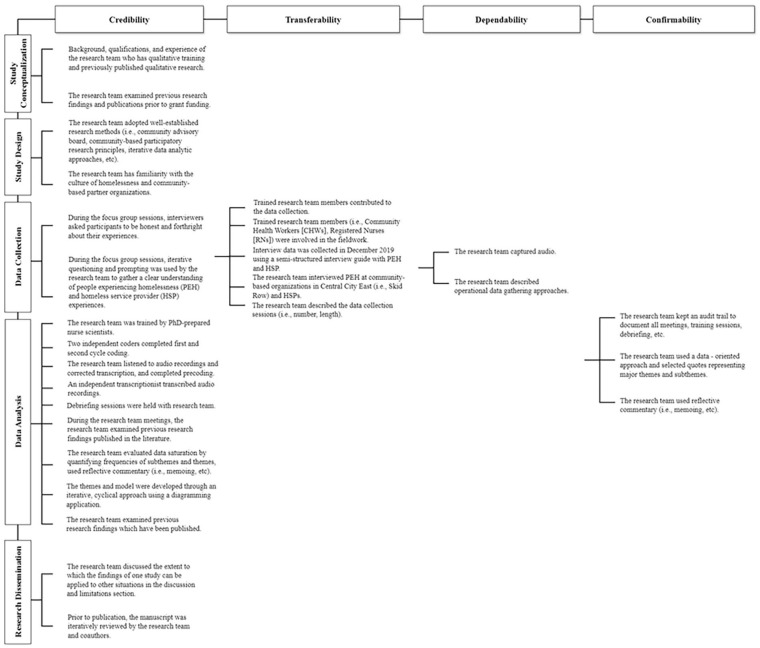
Data analytic approach to ensure the trustworthiness of qualitative data.

Figure 1 provides a detailed description of our data analytic approach which ensured the trustworthiness of data (i.e., credibility, transferability, dependability, and confirmability) (Shenton, 2004). The following section provides a detailed application of how these principles were applied to (a) study conceptualization, (b) study design, (c) data collection, (d) data analysis, and (e) research dissemination.

### Study Conceptualization

To ensure credibility, the research team was composed of investigators who have previous qualitative experience and who examined previous research findings (Shenton, 2004).

### Study Design

To ensure credibility, the research team established well-adopted research methods and developed an early familiarity with the population- and community-based sites (Shenton, 2004).

### Setting and Sample

PEH and HSPs were recruited to participate in this study from several community-based organizations (CBOs) in Skid Row (Central City East, Los Angeles, California). The study team collaborated with the CBO leadership to disseminate study information to interested PEH, and HSP whom they were serving. PEH were enrolled if they self-reported (a) sleeping in the past night in a homeless living situation, (b) being HCV positive as documented by the study sites, (c) history of substance use, (d) ≥18 years of age, and (e) English speaking (Nyamathi et al., 2021). HSPs qualified for the study if they (a) were clinicians (i.e., RNs, nurse practitioners, physicians, physician assistants) or service providers (CHWs, social workers, spiritual advisors), (b) provided educational and social services for PEH, and (c) had ≥1 year working in a PEH clinic, including with PEH treated for HCV infection (Nyamathi et al., 2021).

### Data Collection

PEH and HSPs were enrolled in December 2019 from several sites that serve PEH in Central City East (Skid Row) in Los Angeles, California. To ensure confirmability, we sought to use different types of data source triangulation methods (Carter et al., 2014; Shenton, 2004) including (a) the use of focus group sessions (FGS) and (b) perspectives from PEH and HSP to gain a more thorough understanding of HCV treatment experiences among HCV-positive PEH, and HSPs who work with them to learn how to better support them across the continuum of HCV care to attain and maintain cure. FGS were held in private areas of the community-based sites. To ensure transferability, investigators (BES and AN) trained the research team members to be involved in the data collection. To ensure transferability and dependability, an SSIG was used to guide the FGS which were captured using audio recording and the research team documented data gathering steps. To ensure credibility, interviewers used iterative questioning and encouraged PEH and HSP to be forthright about their lived experiences.

### Data Analysis

**Figure 2.**
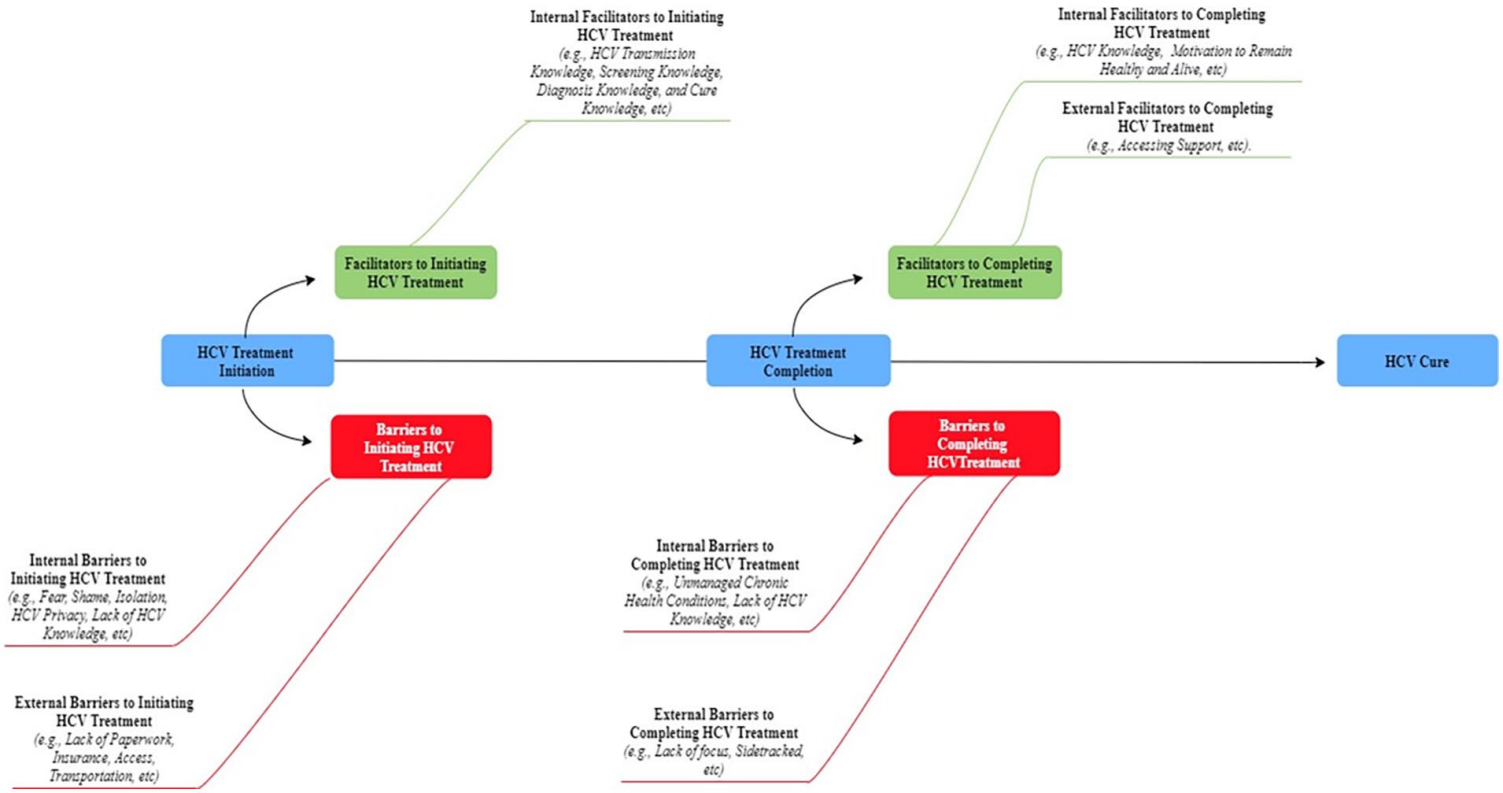
Emergent themes and subthemes on barriers and facilitators across the HCV care continuum voiced by people experiencing homelessness and frontline homeless service providers (*N* = 37).

To ensure confirmability, an audit trail documented the research method steps (Shenton, 2004). To ensure credibility, an independent transcriptionist logged the tapes verbatim. The research team deidentified the transcripts, uploaded them to Dedoose (Dedoose Version 8.3.35, 2020), listened to audio recordings, compared them to one another, and redlined the changes. Afterward, Microsoft Excel was used to organize the data and cycles of coding. To further ensure credibility, a published qualitative researcher (BES) (Salem & Ma-Pham, 2015; Salem, Bustos, et al., 2018; Salem, Kwon, et al., 2018; Salem et al., 2013, 2020, 2021) supervised a research assistant (HA) and collaborated on synthesis, data analysis (precoding, first and second cycle coding), and writing (Saldaña, 2009) for 9 months. To further ensure credibility and confirmability, on a weekly basis, both coders (i.e., BES and HA) debriefed to discuss and reflect on themes and subthemes, beliefs, and assumptions (Shenton, 2004). Thereafter, major themes and subthemes were represented by selected quotes which led to Figure 2 illustration.

### Research Dissemination

To ensure credibility, the findings and the manuscript were peer reviewed by the research team (Shenton, 2004). A full description of our methods is available in our earlier publication (Nyamathi et al., 2021).

## Quantitative and Qualitative Results

Tables 1 and 2 report the characteristics of PEH and HSP which have been published earlier (Nyamathi et al., 2021). As illustrated in Figure 2, major themes emerged such as potential barriers and facilitators across the HCV care continuum which were likely to impact HCV treatment initiation.

**Table 1.** Sample Characteristics of PEH (*N* = 30).

Variable	Mean, *SD*	Range
Age	51.76, 11.49	22–69
Children	3.53, 2.91	1–11
Length of time, homeless	10.24, 12.91	1–59
Not Hispanic/Latino	22	73.3
Hispanic/Latino	8	26.7
*Race/ethnicity*		
Black/African American	15	50.0
Other	11	36.7
White	3	10.0
American Indian/Alaska Native	1	3.3
*Country of birth*		
United States	28	96.6
Other	1	3.4
*Educational attainment*		
Less than ≤12th grade	12	41.4
>12 grade	12	41.4
College/other professional school	4	13.8
Graduate school	1	3.4
*Gender*		
Male	19	63.3
Female	11	36.7
*Intimate relationship status*		
Yes	3	10.3
No	26	89.7
*Children*		
Yes	19	65.5
No	10	34.5
*Religion*		
Protestant (Baptist, Methodist)	10	34.5
Other	10	34.5
Catholic	7	24.1
None	2	6.9
*Employed, ever*		
Yes	15	93.8
No	1	6.3
*Sources of income*		
Unemployed	16	55.2
Disabled	12	41.4
Working part-time	1	3.4
*Unemployment income*		
Yes	1	3.4
No	28	96.6
*General relief or assistance*		
Yes	16	55.2
No	13	44.8
*Food stamps*		
Yes	16	55.2
No	13	44.8
*Assistance from friends or family*		
Yes	7	24.1
No	22	75.9
*Other*		
Yes	3	10.3
No	26	89.7

*Note.* PEH = people experiencing homelessness; SD = standard deviation.

**Table 2.** HSP Sample Characteristics (*N* = 10).

Variable	*N*	%
*Ethnicity*		
Not Hispanic or Latino	7	70.0
Hispanic or Latino	3	30.0
*Race*		
White	5	50.0
Other	4	40.0
Black/African American	1	10.0

*Note.* HSP = homeless service provider.

### Theme 1: Internal Barriers Potentially Impacting HCV Treatment Initiation

PEH and HSPs described *internal barriers potentially impacting HCV treatment initiation* which can encompass feelings of fear, shame, and isolation, lack of HCV knowledge, and the importance of HCV privacy. One participant commented,

. . . What [will their] family . . . think of them, how are they going to treat them. That has a lot to do with it because . . . a friend of mine . . . killed himself when he found out he had Hepatitis. (PEH 12, Focus Group 2)

This participant also described,

It is like, they like don’t know how people . . . [will] . . . feel about them, you know, since a lot of people [are] ashamed. Like, I’m ashamed, you know. It’s a lot of people, it’s a lot of my friends and family don’t know that I have [HCV] because I don’t know what they going to think about me . . . So, it’s hard. (PEH 12, Focus Group 2)

PEH also described a lack of knowledge (e.g., HCV transmission, screening, and diagnosis), questioned how HCV was transmitted, and noted there should be more information about HCV and the next steps. One PEH questioned,

If you don’t use heroin or any crystal meth or any drug that you inject, . . . and if you have unprotected sex, you can get it [Hepatitis] if that person is carrying it . . . you can get it from them? (PEH 8, Focus Group 1)

One participant shared how people may not disclose they have Hepatitis. One participant commented,

Don’t know they [homeless] have it and people don’t check themselves out like they supposed to. So, that’s why some people have it and don’t treat themselves. So, sometimes they don’t know what they have [HCV], you know. (PEH 2, Focus Group 1)

Another participant shared a lack of knowledge that an HCV cure exists within the community.

. . . I don’t know how they treat it now . . . I think that, you know, we need more information about it out there, you know what I mean. I mean there’s no billboards, no signs, no nothing out here. People can just pass by and see it, you know . . . (PEH 15, Focus Group 4)

PEH and HSPs also described *external barriers potentially impacting HCV treatment initiation* including barriers to accessing and linking to HCV care due to lack of paperwork (*e.g., birth certificates, hospital records*), insurance, cost, provider access (*e.g., primary care physician*), lack of available shelter space (*e.g., beds*), and lack of transportation. One HSP noted that in the recuperative setting, a large barrier was the lack of primary care physicians; furthermore, there were challenges with PEH attending follow-up visits or obtaining their prescriptions. Interestingly, some PEH described that they were not encouraged to access treatment and/or did not know how to access treatment. Another HSP said,

Unless it’s been like diagnosed and it’s treatable and then we follow-up like specifically with the diagnosis. It’s sort of tracking down documents, tracking down birth certificates or tracking down even hospital records, has been a difficulty from my end . . .(HSP 3, Focus Group 4)

### Theme 2: Internal Facilitators Potentially Impacting HCV Treatment Initiation

PEH described *internal facilitators potentially impacting HCV treatment initiation including* having HCV knowledge related to transmission (e.g., blood transfusion, sex, sharing drug paraphernalia), testing and diagnosis, access, and cure. One participant said,

Yes, I heard that . . . sharing crack pipes. If I have saliva and I passed my pipe . . . and he has a cut and I have [HCV], I can give it to him. (PEH 1, Focus Group 1)

Another participant shared how they think they got HCV,

I think that’s how I caught mine is in the hospital when I had surgery. I had a spleen removed and I believe it was during surgery the utensils they used or something where it could’ve been through a blood transfusion. (PEH 15, Focus Group 4)

Facilitators to HCV treatment initiation include knowledge that an HCV cure exists. Some PEH described that they learned of the HCV cure when people shared that an HCV cure existed while others were doubtful that an HCV cure existed. One participant shared how she learned about a cure. She said,

. . . And then she had shared with us in a meeting about how she was taking this cure and that it worked for her. (PEH 12, Focus Group 2)

### Theme 3: Internal Barriers Potentially Impacting HCV Treatment Completion

PEH and HSP described *internal barriers potentially impacting HCV treatment completion* including a history of unmanaged chronic health conditions (e.g., HCV, mental illness, and addiction). One participant reflected,

. . . the first barrier was mental illness . . . untreated or undiagnosed mental illness. Drug addiction also can play a small factor in that because it’s very difficult to be consistent with treatment if you’re continually using. So, getting some clean time, that was important. Transportation of course, was one. (HSP 3, Focus Group 4)

PEH also discussed *external barriers potentially impacting HCV medication completion*, including getting sidetracked by friends, substance use, and the need for support. One PEH reflected,

We got drugs. We got people. We got TV. We got radio and we know what our priorities are. But the flesh get weak, okay. And we need some support . . . (PEH 8, Focus Group 1)

One PEH described,

I think, well, I have [HCV] and I know about it, and I’ve been offered the treatment and I could have did the treatment already and they told me not to do it. (PEH 21, Focus Group 4)

This participant continued to share,

. . . And I didn’t do it but now I’m thinking about it because I don’t want [HCV] for the rest of my life. And so, I think it’s different for anybody else. It affects everybody else different, the sickness and the treatment, you know . . . (PEH 21, Focus Group 4)

### Theme 4: Internal Facilitators Potentially Impacting HCV Treatment Completion

PEH described *internal facilitators potentially impacting HCV treatment completion*. One participant explained how they knew the ways that someone could be reinfected with HCV due to substance use. He expressed,

. . . Well, I know of one person . . . she completed the so-called cure, and she got it back. But come to find out, from what I’ve been told, the only way that you can get it back with this cure is to use. (PEH 12, Focus Group 2)

One PEH noted they had hepatitis since the 1980s, while another participant shared that they were tested and received medicine (e.g., injections) for an entire year. One participant said,

I guess it’s still there dormant but so far . . . it’s not detectable, I guess. I don’t know. (PEH 15, Focus Group 4)

PEH discussed *external facilitators potentially impacting HCV treatment completion* including accessing support such as fellowship with others (*e.g., family, 12-step meetings*) and tangible support including incentives (*e.g., money*), convenience, encouragement, the need for HCV-positive people to come forward, and having outreach workers bringing medication to them. Some participants shared that family could help provide the motivation needed to complete HCV treatment. Another participant noted the importance of having HCV support meetings, like those of Alcoholics Anonymous, Narcotics Anonymous, and HIV groups. While another participant noted the importance of accessibility. He said,

It helps [if they can] . . . get [HCV] treatment . . . close by where they have their priorities straight . . . And like I said, it’s convenient. (PEH 1, Focus Group 1)

A CHW echoed the importance of convenience and providing encouragement.

Yes, and to be encouraged . . . so that we can come out to them, because for a lot of the homeless, it’s hard for them to get started and get moving for the day. So, if [we] were there to bring them the medication, compensate them as well, I think that would be great. (HSP 1 [CHW], Focus Group 1)

Another external HCV treatment completion facilitator included providing encouragement, along with addressing housing and community insecurity. One Chaplain noted,

I think that if at least for our side of what we work on in program, it’s just helpful to have the community support and the emotional support . . . [as] there’s so many things to have to do in any given day or week that you just get exhausted. And so, just to have someone who’s just there to support you to encourage you to keep taking it [the HCV treatment], remind you of the reason why you’re taking it and the hope for it [to cure HCV] . . . (HSP 4, Focus Group 4)

Another Chaplain noted,

Yeah, but like here in the facility, with we’ve already taken care of your housing insecurity . . . Then and I can take care of your community insecurity because it’s very difficult to do it alone. You have very little hope if you’re isolated. So, moving people out of isolation into community made it much easier to then walk-through treatment and coverage. (HSP 3, Focus Group 4)

PEH and HSPs (*e.g., Chaplains, RNs*) discussed *external facilitators potentially impacting HCV treatment completion* including accessing support during HCV treatment (e.g., making appointments, accompanying clients, available and frequent contact, medication reminders, following up on appointments, support meetings, role modeling, housing, transportation). One participant shared,

. . . what I saw was helping folks who were . . . able to address the barriers of consistently showing up for treatment. I helped walk someone with the pill form. It was just hope that I’m going to get better in the midst of taking it every day. Like, and it actually will work and I will not have to feel just like yucky. And that even if I may not feel that great while I go through it, because there are some, you know, side effects, that they knew that they weren’t alone and they had hope that this will not just be something they’ll manage but will be cured. (HSP 3, Focus Group 4)

Commenting on the importance of accompanying participants to appointments, an RN shared,

. . . What I’ve noticed with a lot of our clients is . . . even if they do go see their [primary care provider] (PCP), when they come back, they don’t have their paperwork. They lose their paperwork. Or if we ask, you know, what happened at the appointment, they’ll say nothing. Nothing happened. Nothing changed. Nothing’s new. So, what we’ve been doing to help the client is actually attending with them . . . that really helps . . . (HSP 1 [RN], Focus Group 3)

Another participant expressed,

. . . Be a role model to the ones that have it and inspire them to do the right thing for themselves or anybody else if they want to stay. (PEH 13, Focus Group 2)

PEH described the importance of housing and its interrelationship with HCV treatment. One participant shared,

If I could get housing, then I would . . . the treatment. (PEH 17, Focus Group 4)

## Discussion

HCV is highly prevalent among PEH and necessitates multidisciplinary intervention; however, few studies have developed HCV medication adherence interventions for PEH. This paper is part of a two-part qualitative series (Nyamathi et al., 2021) which has a common purpose; however, each paper uniquely contributes to the understanding of HCV treatment experiences among HCV-positive PEH and HSPs to tailor the “I am HCV Free” intervention for PEH. The first paper aimed to understand the perspectives of HCV-positive PEH, and HSPs on the design and delivery of an HCV intervention program (Nyamathi et al., 2021). However, the following paper focuses on the perspectives of PEH and HSP on barriers and facilitators that have the potential to impact the HCV care continuum including HCV treatment initiation, completion, and cure.

PEH described internal (fear, shame, etc.) and external barriers and facilitators that have the potential to impact HCV treatment initiation. Identifying feelings of shame and fear, and creating supportive and inclusive messages, along with helping PEH prioritize health or tap into their desire to be healthy can lead to successful HCV treatment initiation. While there are limited evaluations of peer programs (Crawford & Bath, 2013), peers may be able to successfully address HCV-related myths (Batchelder et al., 2017) and mitigate shame and fear among PEH.

Some PEH had knowledge of HCV transmission including how sharing drug paraphernalia leads to HCV transmission and conjectured how HCV was transmitted to them. Extant research reveals that HCV education improves HCV knowledge and therapy (Lubega et al., 2013); however, low HCV treatment knowledge and variability between service sites exist (Fokuo et al., 2020) which may compromise HCV treatment initiation.

PEH shared barriers to HCV diagnosis which can also impact HCV treatment initiation including not knowing they had a disease and guessing about the virologic detectability. Translating health knowledge, considering health literacy among this vulnerable population, and following up with HCV test results and diagnosis are critical.

Concerns were expressed by PEH and HSP related to barriers to HCV linkage to care which has the potential to impact HCV treatment initiation. There was a sense among participants that facilitators to HCV treatment linkage included assisting PEH with paperwork, accompanying clients to appointments, medication completion, and role modeling. Previous research has found that insurance (e.g., high out-of-pocket costs, lack of insurance, and excessive paperwork) was one of the most important barriers to HCV care (Fokuo et al., 2020).

PEH also discussed external barriers and facilitators to HCV treatment completion. Some internal barriers PEH experiences include a history of unmanaged chronic health conditions, such as mental health conditions and drug addiction. An external barrier that has the potential to impact HCV treatment completion includes how individuals can either serve as a distraction or a facilitator of HCV treatment, such as providing fellowship and support. These findings suggest the need to consider the importance of managing concurrent chronic health conditions and providing the necessary support to support HCV medication adherence.

Potential external facilitators to HCV treatment completion include providing incentives and convenient locations for HCV treatment delivery. HCV care continuity is a critical issue that requires system-level commitment and support, including shelter partnerships and stable housing which has been corroborated by other studies (Fokuo et al., 2020). Accompanying clients to appointments was also a critical issue and others have noted how peer support can enhance mutual trust and social support and reduce stigma (Batchelder et al., 2017). Furthermore, participants described mixed information related to existing knowledge of HCV cure or not having much knowledge. Future research should provide education about pharmacologic and treatment modalities for HCV infection.

### Integrating PEH and HSP Views to Further Refine the “I am HCV Free” Intervention

Based on these findings, the “I am HCV Free” intervention was further developed to be delivered by a nurse and CHW who would lead eight HCV education sessions which focus on (1) orientation, liver basics, and structure; (2) hepatitis causes and risk factors; (3) the effects of alcohol and drug use on the liver; (4) risk factors for HCV transmission; (5) HCV medication completion; (6) ongoing HCV medication adherence; (7) HCV chronic disease self-management to remain HCV free; and (8) lifelong chronic disease self-management to remain HCV free. During the weekly sessions, the RN/CHW team would also link clients to HCV care, refer participants to other health and social services, accompany them to appointments, and monitor medication side effects and adherence.

### Limitations

Despite these promising results, these findings have four main limitations. First, these findings are limited to Skid Row, Los Angeles, for those between 22 and 69 years of age. Second, this sample had a disproportionate number of men as compared to women and HSPs. Third, data saturation and an in-depth understanding of HSP experiences were challenged by the study’s disproportionate sample of PEH and HSPs. Fourth, some participants may be unaware of the services that are provided within the organization and community.

## Conclusions

Notwithstanding these limitations, this study has explored perceptions of HCV treatment experiences among HCV-positive PEH and HSPs and lays the groundwork to further tailor the “I am HCV Free” intervention with particular attention to the delivery and content. The findings from this study add to the growing body of research surrounding RN/CHW interventions but make several unique contributions to the current literature. First, the results of this investigation have enhanced our understanding of barriers and facilitators that have the potential to impact the HCV care continuum among PEH. Second, these findings have significant implications for the delivery of HCV interventions for PEH and how they can be tailored for PEH across the continuum of HCV care.

However, there are still unanswered and lingering questions; for instance, what are the effects of the “I am HCV Free” intervention on PEH, HSP, and the broader community? Can the RN/CHW model successfully support this population to remain HCV-free? Given that many PEH have other chronic physical and mental health conditions, should the RN/CHW model be tailored for both physical and mental health chronic disease self-management and HCV care coordination? Given that PEH can be reinfected with HCV after SVR, what kind of messaging and intervention integration can be developed to help PEH maintain HCV-free status? Should primary, secondary, and tertiary-level approaches be further integrated into an HCV intervention for PEH? How can the outcomes of the “I am HCV Free” intervention be translated to different settings and geographical locales? Aligned with the WHO’s goals to eliminate HCV infection (WHO, 2022), our findings suggest that individual and structural-level intervention tailoring is needed for PEH to maintain and attain HCV cure. Considerably more work will need to be done to gain a greater understanding of the viability of these approaches and how each can be tailored to different settings and geographic regions among PEH across the HCV care continuum for them to successfully attain and maintain HCV cure.

## Supplemental Material

sj-docx-1-cnr-10.1177_10547738241273104 – Supplemental material for Exploring the Perspectives of Unhoused Adults and Providers Across the HCV Care Continuum Supplemental material, sj-docx-1-cnr-10.1177_10547738241273104 for Exploring the Perspectives of Unhoused Adults and Providers Across the HCV Care Continuum by Benissa E. Salem, Helena Almeida, Sarah Akure Wall, Kartik Yadav, Alicia H. Chang, Lillian Gelberg and Adeline Nyamathi in Clinical Nursing Research
