# Supplementary material for: Exploring the Perspectives of Unhoused Adults and Providers Across the HCV Care Continuum
Source: Clin Nurs Res. 2024 Sep 9;33(7):519–29. doi: 10.1177/10547738241273104 (PMC11421191; doi:10.1177/10547738241273104)
Supplement: sj-docx-1-cnr-10.1177_10547738241273104 – Supplemental material for Exploring the Perspectives of Unhoused Adults and Providers Across the HCV Care Continuum [file sj-docx-1-cnr-10.1177_10547738241273104.docx]

Supplemental Material

The semi-structured interview guide (SSIG) prompted questions such as (1) “What health services you have sought in the last few years?”; (2) “If you ever had a time when you had a health problem but did not seek care for it, what got in the way?”; (3) “What would you say are the biggest barriers to completing the therapy (HCV)?”; and, (4) “What would you recommend to successfully engage PEH to complete the testing process?”
